# Perspectives on the Application of Nanomaterials in Medical and Dental Practices

**DOI:** 10.7759/cureus.43565

**Published:** 2023-08-16

**Authors:** Samruddhi Rathi, Amit Reche, Nutan Dhamdhere, Akarsh Bolenwar

**Affiliations:** 1 Public Health Dentistry, Sharad Pawar Dental College, Datta Meghe Institute of Higher Education and Research, Wardha, IND

**Keywords:** nanocomposites, medicine, tumour, encapsulation, fluorescence, nanoparticles

## Abstract

A new field of study called nanotechnology concentrates on manipulating matter at atomic and molecular levels. Modern medicine may benefit tremendously from developments in the field of nanotechnology, and as a result, nanomedicine has emerged as a key location of education in the specific area of nanotechnology. This article aims to describe nanotechnology's possible applications in therapeutics. Nanotechnology and nanomedicine have allowed for the development of new dental materials that are stronger, more resistant to microbial seeding, etc. Other examples include high-strength denture bases, antimicrobial dental glue, aesthetic restorative materials comprised of small particles, and interface adorning for dental posts. Nanotechnology has been perfectly utilized in the medical industry for tissue engineering, biosensors, nanoscale diagnostic tools, and medication delivery using nanoparticles.

## Introduction and background

"Nano" is a Greek term that means "micro". Current nanotechnology research is focused on developing a dependable and environmentally friendly chemical approach for the biogenic production of nanomaterials [1]. Nanoparticles bind strongly to other materials or one another due to their large surface-free energy (agglomeration). These effects might be used in nanoparticle bulk applications [2]. Due to their unique chemical and biological properties, as well as their many applications in the domains of optics, electronics, biomedicine, magnetics, mechanics, catalysis, energy research, and other areas, nanostructured materials are receiving a lot of interest [3]. It enables the integration of traditional microtechnology with a cellular science approach in real-world applications. Research is now being done on several therapeutic nanotechnology uses, including molecular imaging, drug administration, and disease detection [4]. Some possible advantages of this trend include the following: 1) the metabolic properties of the sick tissues can be exploited to target treatments [5]; 2) different nanotechnology-based products can form in larger amounts than traditional pharmaceuticals [6]; 3) poor elimination of lymph nodes and higher blood flow in tumors enhance the transfer and persistence of nanosystems in tumors or damaged tissues [7,8]; 4) selectively locating inflamed regions using nanotechnologies is possible [9]; and 5) bypassing the barrier that separates blood from the brain (meninges), nanostructures may be used to effectively transmit or convey essential drugs throughout the cerebral [10,11]. Therapeutic efficacy and safety are improved when medication is absorbed into nanotechnology because it changes how it circulates organs and tissues and leads to more efficient delivery of physiological chemicals [12,13].

A field of study has been dubbed "nanodentistry," which is described considering the fields of science and expertise of detecting, oral and dental disease treatment, as well as avoidance disorders, pain relief, and dental health preservation and improvement utilizing nanostructured materials [14]. The antibacterial capabilities of silver nanoparticles (AgNPs) have been successfully used in a variety of medicinal applications [15]. The purpose of using AgNPs is to minimize microbial colonization of dental biomaterials, thereby enhancing oral health [16].

## Review

Publications on connected themes were researched and monitored using databases such as ResearchGate, PubMed, Google Scholar, and MedlinePlus. Quantitative data were obtained and analyzed more thoroughly. In addition to web-based resources, content from the WHO was used to conduct the research. In addition, this paper was recommended to Scopus and PLOS ONE.

Nanoparticles in medical practice

Such a sophisticated biological system implementation of such an approach will unquestionably alter the fundamentals of disease detection, therapy, and avoidance in the coming years. Some of these uses are covered in the following sections.

Utilization of Nanostructures for Diagnostics and Medical Diagnosis

Improved luminescence indicators for detection and evaluation reasons are the principal and early uses of nanomedicine. Traditional fluorescent markers need complicated lasers that coordinate with shades that can only be used once before fluorescence fades and have limited discriminating capability owing to blood reddening. Fluorescent nanoparticles, such as "quantum dots," probes encapsulated by biologically localized embedding (PEBBLEs), and perfluorocarbon particles, may be able to address these problems. Nanoparticles can also be employed to tag several biomolecules at the same time, both within and outside the cells, to track disease development. Nanoparticles can be used as magnetic resonance contrast agents in live cells [17].

Through monitoring the attachment of a specific antibody to the illness-associated goal, many current and customary investigations can identify a component or living thing's existence that causes disease. These investigations are frequently accomplished by binding the immunoglobulins using both natural and synthetic hues and utilizing electronic or microscopy with fluorescence to see the messages within the samples. The specificity and viability of the detection methods are frequently constrained by dyes, though. Utilizing semiconductor nanocrystals (also known as "quantum dots"), nanobiotechnology provides a remedy. Compared to normal organic molecules, which more easily degrade, these tiny probes can resist a substantial number of cycles of excitations and light emissions [18].

Drug Distribution Using the Nanoscale

In the field of nanotechnology, nanocrystals are used to supply drugs to particular locations. The required dosage of the medicine is employed in this procedure; possible adverse consequences are greatly reduced since the substance is only dumped within an ominous area. This very targeted technique can help patients save money and have less discomfort. As a result, a wide range of nanoparticles, such as dendrimers and nanoporous materials, are used. For medication encapsulation, micelles made from block co-polymers are utilized. They deliver tiny medication molecules to their intended destination. For the active release of medications, nanoelectromechanical devices are also used. The use of iron nanoparticles or gold shells in cancer therapy is gaining traction. Targeted medication minimizes pharmaceutical expenses and therapeutic expenditures are reduced, lowering the cost of healthcare for patients [19]. Numerous medications with limited permeability that can't be given orally are currently allowed to be utilized in therapy thanks to the application of nanostructures [20,21].

Nanotechnology in Cardiac Therapy

Presently, nanomaterials have presented potential techniques for use in contemporary heart physiology, enabling the biological discovery of fresh opportunities and improvements in the treatment of complicated cardiac issues. These methods have applications in biology, scans, and detection [22]. Additionally, the nanoscale could assist with the identification and description of specific cardiac disease-related pathways that are medically important. It can also be utilized to build the level of atoms in gadgets; this might be molecularly implemented in the structures of biology. The inclusion of these recently created tiny machines may significantly alter various concepts and assumptions about the treatment of serious coronary artery disease. Using nanostructures to solve problems like fragile coatings and gate transparency may also be beneficial. The ability to get specific and ongoing vascular and coronary drug treatments to relieve the symptoms of heat-related illnesses may thus be significantly improved by using this approach [23].

Nanotechnology in Orthopedic Applications

Using tiny materials, such as mini plastics, graphite microfibers, nanotubes, and porcelain, may make it easier for calcium-containing mineral deposits to build up on prostheses. Regarding these results and conclusions, nano-shaped substances symbolize a novel area of study development that could enhance bone cell interactions to improve the adhesion of an implant to the tissues of the bone. This will help to increase the effectiveness of artificial joints while significantly lowering adherence among patients’ issues [4].

Application of Nanotechnology in Cancer

Owing to their microscopic size, microscopic particles might be highly helpful in the treatment of carcinoma, especially in scanning. Outstanding representations of tumor sites may be produced using radiation therapy and quantum entanglement characteristics, which include size-tunable radiation. Particles, especially those used in imaging, can be very useful in cancer research due to their tiny dimensions. Magnification resonance imaging may be utilized to provide beautiful pictures of tumor locations using finite enclosing capabilities with size-tunable photon release, for instance [24]. Nanocircuits are used to create detector diagnostic cards that are capable of discovering and diagnosing cancer in its early stages using just a few drops of a patient's blood and can detect proteins and other indicators left by cancer cells [25].

**Figure 1:**
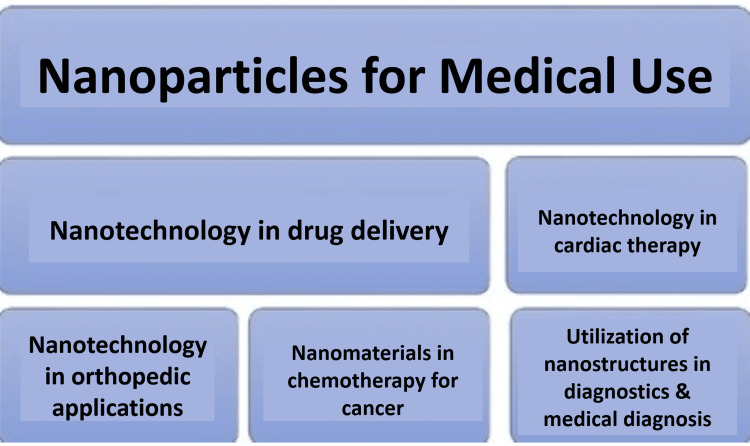
Biomedical applications of nanoparticles

The prospect of using nanomedicine in a variety of medical sectors has increased as a result of ongoing advancements in the field. Research is now being done on its possibilities for use in monitoring and rejuvenating therapies. In terms of assessment, sick colonies may be treated right away before extending to other parts of the body since diseased cells could be found more rapidly, possibly down to the level of the sick cell. Treatment with the use of nanomedicine may also be beneficial for people who have suffered severe accidents or have impaired organ function. Figure 1 shows the biomedical applications of nanoparticles.

Nanoparticles in dental practice

With the advent of nanotechnological research concentrating on the synthesis and use of nanomaterials with superior mechanical properties, dental materials have developed. Manufacturers have included dental components that include nanoparticles (such as gemstones, colloidal silicate, zirconium, and aluminum oxide) to enhance these materials' chemical and physical attributes [26]. Dental composites using nanoparticles may have improved fracture toughness and tooth tissue adhesion [27]. Utilizing ceramic nanoparticles to polish the outermost layer of teeth might be helpful in the prevention of cariogenic damage and bacteria, possibly because microorganisms are more readily removed. This has been tested in vivo on human teeth [28]. As a result, nanoparticles may increase the mechanical qualities of dental restorative materials, like the hardness of the exterior and resistance to erosion [29].

Antimicrobial Dental Nanomaterials

Fundamental issues in dental care must be taken into account when developing AgNPs, including nanocrystal access to a memorial microbiome that has polar opposite connection dimensions and effectiveness, as small particles larger than 50 nm cannot pass through the biofilm's barrier [30]. Nanocrystals with an unfavorable valence exhibit a hard time spreading throughout biofilms, presumably because carboxylate and phosphoryl units are found on bacterium surfaces, which make the cell surface electronegative.

Nanocomposites

Dental materials made of polymers and nanotechnology incorporating nanocrystals now offer the best practical likelihood, based on the diversity of nano-based dental materials presented here. When compared to dental resin composites containing microparticles, utilizing nanoscale, some of these dental products are currently on the market, enabling the incorporation of additional plugs. Even though several of these dental materials have already been marketed, concerns remain regarding the inhalation of nanomaterials that are generated during mechanical procedures, including sculpting, buffing, and dissolving restorations comprised of these nanocomposites [31]. Recently, large ionic-density nanogel particles have been put to use in dental resin composites to reduce shrinkage during polymerization and tooth structure loads without compromising the plastics' other crucial properties [32,33]. Additionally, the creation of water-compatible, extensively bonded hydrocarbons that extend the longevity of phase-separated, super-hydrophilic coupling adhesives has been made possible by the use of various hydrophilicity nanogels [34].

Nanomaterials Combined with Alloys and Cements

Therapeutic restorative materials are required for non-traumatic and noninvasive cosmetic dentistry. To achieve this, fluorinated apatite nanoscale and titanium dioxide nanoparticles have been created and studied as potential curative cavity restorations that might avoid and/or alleviate cavities [35,36]. In neurological damage studies comparing the results of easily accessible studies, the potential for biocompatibility of dental materials containing this substance, nanoparticles, has been investigated using traditional ceramic cement and nano-based strengthening for root-end filling in endodontics. When human mesenchymal stem cells are exposed to a type of nanocement, they exhibit similar properties (i.e., biocompatibility) to mineral trioxide aggregate (MTA) [37].

Nanomaterials for Therapeutic Dentistry

While showing strong effectiveness against oral carcinomas of squamous cells, antitumor nanostructures containing cisplatin, dispersed on polyethylene glycol-poly (glutamic acid) caused significantly less renal damage than iron itself [38]. Additionally, in monolayer-cultured human oral squamous cell carcinoma, lipid nanoparticles that are solid in shape function as efficient carriers for hydrophobic or water-labile irradiation, providing the benefits of improved stability of the medication, protection against degradation by proteolytic enzymes, and ongoing emission of the absorbed elements [39].

Biomodulation of Dental Tissues Through Nanotechnology

**Figure 2:**
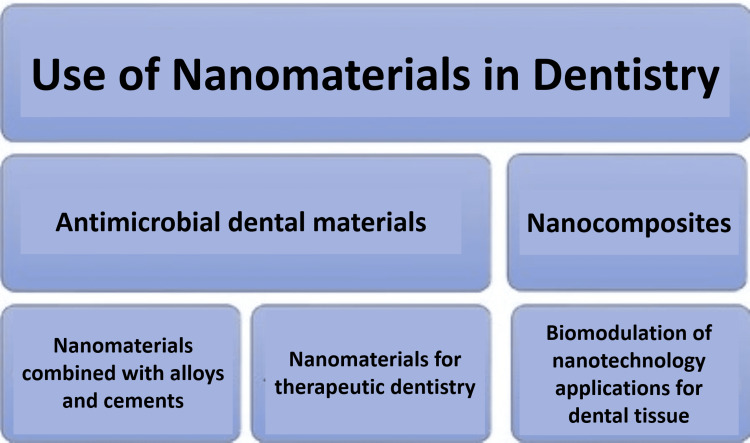
Use of nanomaterials in dentistry

Dental supplies of many types can be utilized to create nanotechnology formations. When resin-bonding solutions with certain functional monomers are used, for instance, linking the resin-dentin user experience, nano-layers of calcium or hydroxyapatite may self-assemble [40,41]. For the remineralization of dentin collagen, many functional additives that can release calcium and phosphate are now available. Under the right pH and temperature circumstances, these ions are prone to precipitating again into apatite and/or octa phosphate of calcium, which are more sophisticated limestone phosphor chemicals. Biomimetic analogs of dentin with no collagenous proteins have been shown to stabilize amorphous calcium phosphate as 'polymer-stabilized aqueous intermediates.' It was shown that these liquid-like amorphous phosphates of calcium nanodroplets might enter the underground water reservoirs of filaments of cartilage, causing gelatin filament intrafibrillar restoration of minerals [42]. Figure 2 shows the use of nanomaterials in dentistry.

Use of Nanomaterials in Periodontics

All of these materials might potentially be created in the future for periodontal medication delivery systems. Drugs can be added to nanospheres made of biodegradable polymers, which enables timed drug release when the nanospheres break down. The recent invention of Arestin, in which tetracycline is integrated into micro-spheres for local medication delivery to a periodontal pocket, is a nice illustration of how this technology may be developed [43]. Drug molecules can be enclosed in nanoparticles to be delivered to specific regions of the body afflicted by periodontal disease. By placing the regulated amount of medicine in the vicinity of the region of interest, this strategy can lessen dosage-related adverse effects [44]. To create room for genuine periodontal tissue regeneration, guided tissue regeneration for periodontal defect healing uses a barrier membrane surrounding the periodontal defect to prevent epithelial downgrowth and fibroblast transgrowth into the wound site [45].

Future scope of nanomaterials 

Nanomedicine is a common use of nanotechnology in the medical profession. Future applications of certain nanomaterials might include tissue engineering, biomedical implants, pharmaceutical items, advanced imaging and technique, and diagnostic instruments. Nanomaterials provide a cutting-edge diagnostic platform for genetic illnesses since they are non-invasive, easy to use, portable, and affordable. Additionally, several nanomaterials have been created and integrated with target molecules to provide therapeutic benefits that are both selective and useful for molecular imaging.

Limitations of the review 

Limited information is available regarding the uses of nanomaterials in the medical field. Cost is a barrier to using nanomaterials in the medical industry. As a result, the toxic effects of the nanoparticles endanger both people and the environment. Not many articles are published regarding this topic, and the results are tested on a limited population. Awareness about nanomaterials is very low.

## Conclusions

In this review article, most of the advances in medicine and dentistry using nanomaterials are explained. Nowadays, the use of nanomedicine is providing advancement in all fields. Nanobiotechnology has a wide range of medical applications. Drug delivery methods, for example, are simply the beginnings of something new. Many diseases that currently have no therapies may be healed in the future, thanks to nanotechnology. Even though the expectations and benefits of nanobiotechnology in medicine are numerous, including potential advantages that are continually enumerated, the security of nanomedicine is still a work in progress. Nanotechnology's use in medical therapies necessitates a thorough assessment of its risks and safety concerns. Many types of dental materials, comprising reinforces and synthetic resins It is possible to substitute lost dental components that have been developed using nanotechnology and are now accessible for clinical usage. To achieve fantastic ongoing psychological and mechanical traits like superior resilience, rigidity, robustness, and durability against wear while mimicking the appearance of tooth structures, ceramic and on-the-market accessible synthetic resins now contain silica-rich crystal nanocrystals. In the years to come, the field of nanotechnology will play a significant role in medicine, offering ground-breaking opportunities for early identification of illness, testing, and treatment, as well as boosting medical care and individuals' motor skills. It will also enable precise and successful therapy specifically tailored to patients.
